# Identification of key proteins in early-onset Alzheimer’s disease based on WGCNA

**DOI:** 10.3389/fnagi.2024.1412222

**Published:** 2024-10-09

**Authors:** Dazhi Li, Yaxin Wang, Jinliang Wang, Qiqiang Tang

**Affiliations:** Department of Neurology, The First Affiliated Hospital of USTC, Division of Life Sciences and Medicine, University of Science and Technology of China, Hefei, China

**Keywords:** early-onset Alzheimer disease, WGCNA, proteome, biological process, therapeutic target

## Abstract

**Introduction:**

Early-onset Alzheimer’s disease (EOAD) is sporadic, highly heterogeneous, and its underlying pathogenic mechanisms remain largely elusive. Proteomics research aims to uncover the biological processes and key proteins involved in disease progression. However, no proteomic studies to date have specifically focused on EOAD brain tissue.

**Method:**

We integrated proteomic data from brain tissues of two Alzheimer’s disease (AD) cohorts and constructed a protein co-expression network using weighted gene co-expression network analysis (WGCNA). We identified modules associated with EOAD, conducted functional enrichment analysis to understand the biological processes involved in EOAD, and pinpointed potential key proteins within the core modules most closely linked to AD pathology.

**Results:**

In this study, we identified a total of 2,749 proteins associated with EOAD. Through protein co-expression network analysis, we discovered 41 distinct co-expression modules. Notably, the proteins within the core module most closely linked to AD pathology were significantly enriched in neutrophil degranulation. Additionally, we identified two potential key proteins within this core module that have not been previously reported in AD and validated their expression levels in 5xFAD mice.

**Conclusion:**

In summary, through a protein co-expression network analysis, we identified EOAD-related biological processes and molecular pathways, and screened and validated two key proteins, ERBB2IP and LSP1. These proteins may play an important role in the progression of EOAD, suggesting they could serve as potential therapeutic targets for the disease.

## 1 Introduction

Alzheimer’s disease (AD) is one of the most prevalent forms of dementia globally. When symptoms manifest before the age of 65, the condition is classified as early-onset Alzheimer’s disease (EOAD), which is relatively rare, accounting for only 5% of all AD cases. In contrast, the majority of cases, where symptoms develop after the age of 65, are classified as late-onset Alzheimer’s disease (LOAD) (Gauthier et al., 2022). There are notable differences between EOAD and LOAD in terms of clinical symptoms, genetics, neuroimaging, and pathological changes (Sirkis et al., 2022). The clinical heterogeneity observed in EOAD underscores the importance of dedicated research on this subgroup. Understanding the underlying pathogenesis of EOAD could also facilitate the development of new therapeutic agents (Fang et al., 2022).

Weighted gene co-expression analysis (WGCNA) is a specialized bioinformatics methodology used to investigate biological networks, signaling pathways, and variations in cell types within human tissues (Miller et al., 2008). By linking co-expressed protein modules to clinical features, WGCNA helps to elucidate the relationship between these modules and specific clinical characteristics. Moreover, it can identify key drivers of disease onset within the proteins most strongly associated with these co-expressed modules (Barabási et al., 2011). Consequently, WGCNA is widely employed to pinpoint potential biomarkers or therapeutic targets (Yue et al., 2017).

**Figure 1:**
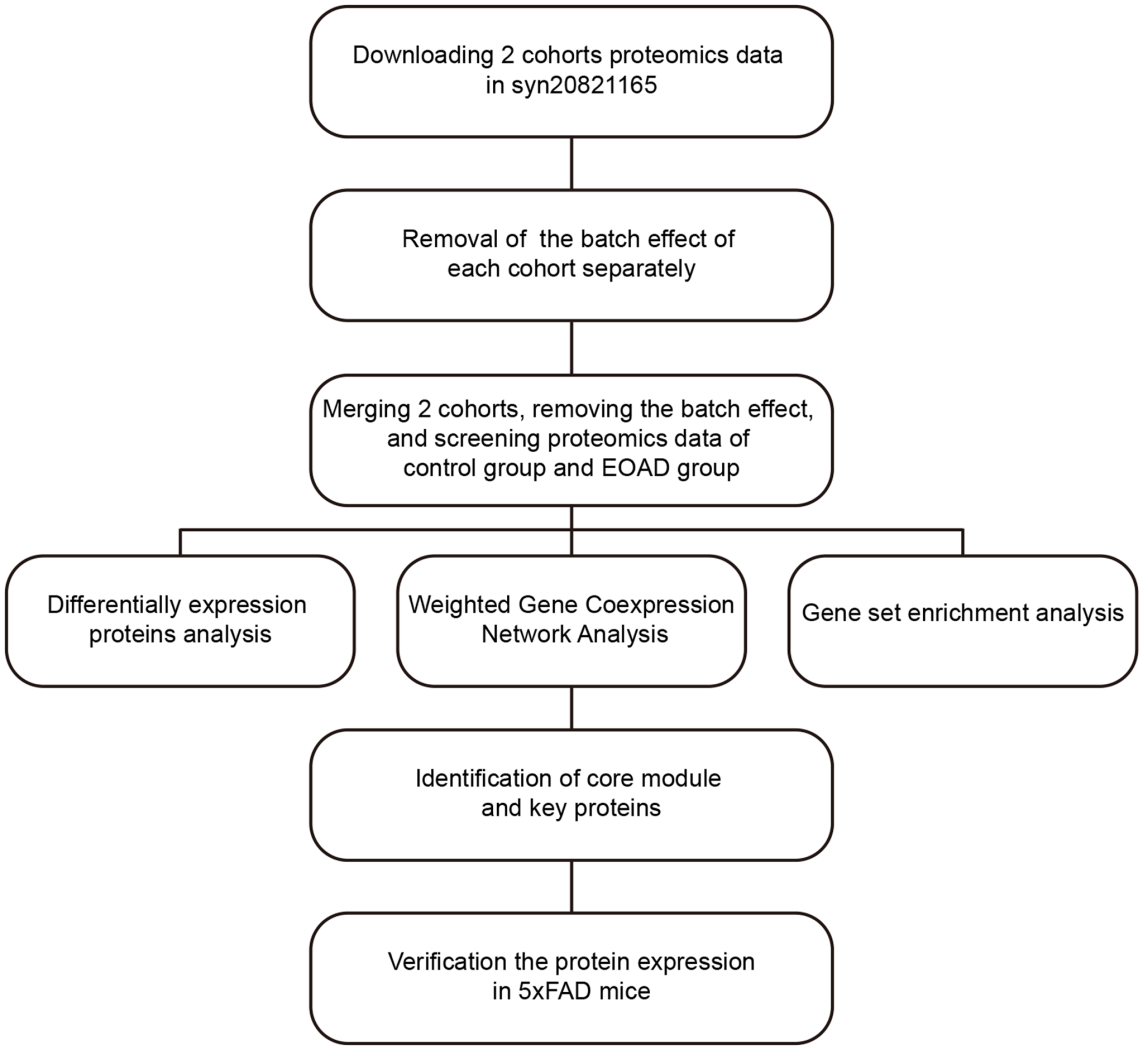
Workflow to identify the key proteins of EOAD.

Due to the rarity of EOAD and the strict requirements for collecting human brain tissue within hours postmortem, obtaining brain samples from EOAD patients is extremely challenging. In this study, as outlined in the workflow chart in Figure 1, we integrated and analyzed proteomic data from two AD cohorts at Emory University to conduct a preliminary investigation of molecular changes in the brains of individuals with EOAD. After adjusting for batch effects and covariates, we identified 2,749 EOAD-related proteins. We then constructed an EOAD brain protein co-expression network, comprising 41 protein modules, and performed correlation analyses with AD pathology and biomarkers from various brain cell types. This approach allowed us to explore the biological processes and signaling pathways enriched in the protein modules associated with EOAD. Furthermore, we identified two key proteins within the modules most significantly linked to EOAD and validated their potential as therapeutic targets in an AD mouse model.

## 2 Methods

### 2.1 Controlling for batch effects in proteomic datasets

In this study, clinical information and protein expression profiles for two cohorts were downloaded from Synapse (Synapse ID: syn20821165). We used the TAMPOR method to eliminate technical batch differences within the proteomic data while preserving meaningful biological variations in protein expression levels. This method normalizes samples within selected batches or cohorts to a central tendency, ensuring consistency across the data (Johnson et al., 2020). The TAMPOR algorithm is fully documented and available as an R function, which can be accessed at https://github.com/edammer/TAMPOR. Initially, we removed intra-cohort batch effects from each cohort separately. We then merged the protein expression profiles of the two cohorts based on UniProt IDs and subsequently eliminated inter-cohort batch effects.

### 2.2 Statistical analysis

Statistical analysis was conducted using SPSS (version 26). Student’s t-test was used for pairwise comparisons of continuous variables. Chi-square test was employed for between-group comparisons of categorical variables.

### 2.3 WGCNA

The protein co-expression network was constructed using the “WGCNA” package in R, where the Pearson correlation coefficient (cor) was calculated to determine the correlations between proteins. To ensure a scale-free network, a soft-thresholding power (*β*) of 13.5 was selected to transform the similarity matrix into an adjacency matrix. Subsequently, a topological overlap matrix was built to measure the average network connectivity for each protein. Based on the parameters (deepSplit set to 2 and minModuleSize set to 30), proteins with similar expression profiles were divided into different modules using the dynamic tree cutting method, with a cutHeight value set to 0.9. Hierarchical clustering was used to construct a dendrogram, calculate the correlation between module eigengenes and traits, and thereby select module eigengenes (MEs). Modules with the highest correlation to AD pathology were further analyzed as core modules.

### 2.4 Enrichment analysis

Gene Set Enrichment Analysis (GSEA) was conducted using the “clusterProfiler” package in R (Yu et al., 2012). Functional clustering analysis was carried out using Metascope (Zhou et al., 2019). Fisher’s exact test was employed with a human cell type marker list to determine cell type enrichment, and adjustments were made using the Benjamini-Hochberg procedure (Johnson et al., 2022).

### 2.5 Experimental animals

All animal experiments were approved by the Animal Ethics Committee of the First Affiliated Hospital of University of Science and Technology of China (2024-N(A)-30). 5xFAD transgenic mice and their littermate wild-type mice at the ages of 3, 6, and 10 months were selected for the study. All mice were housed in groups of 5–6 per cage under aseptic conditions with controlled temperature (22–25°C), and a 12-h light/dark cycle alternation.

### 2.6 Sample processing and immunoblotting experiments

The mouse brain tissue was ground into powder in liquid nitrogen, weighed using an analytical balance, and then proportionally added to RIPA lysis buffer. The mixture was ground with a pestle for 100 times, followed by sonication for 3 min, and centrifugation at 12,000 g for 30 min. Loading buffer and DTT were then added to prepare the samples for immunoblotting.

The protein lysates were separated on a sodium dodecyl sulfate-polyacrylamide gel electrophoresis (SDS-PAGE) gels and then transferred onto a polyvinylidene fluoride (PVDF) membranes (Millipore, USA). The membranes were blocked with 5% non-fat milk at room temperature for 1 h to prevent nonspecific binding. Subsequently, the membranes were incubated overnight at 4°C with primary antibodies as follows: mouse anti-APP-6E10 (1:2,000, BioLegend, Cat# SIG-39320), mouse anti-LSP1 (1:1,000, Santa Cruz Biotechnology, Cat#sc-271137), rabbit anti-Erbin (1:500, Novus Biologicals, Cat#NBP2-56104), mouse anti-GAPDH (1:10,000, Milipore, Cat#MAB374), mouse anti-*β*-actin (1:10,000, Proteintech, Cat#60008-1-Ig). The membranes were then washed three times for 10 min each with TBST solution to remove unbound antibodies. Subsequently, they were incubated with secondary antibodies (HRP-conjugated anti-rabbit or anti-mouse IgG, 1:5,000, Proteintech) for 1.5 h, followed by washing and chemiluminescent detection.

## 3 Results

### 3.1 Data collection

Given the rarity of EOAD and the difficulties in obtaining brain tissue specimens, we conducted a preliminary investigation of molecular changes in the brains of individuals with EOAD by integrating proteomic data from the dorsolateral prefrontal cortex of two AD cohorts at Emory University. The AD cases in these cohorts were diagnosed according to the NIA-Reagan criteria for AD (Hyman and Trojanowski, 1997). Protein quantification was performed using multiplex tandem mass tag technology. Before analysis, we used the TAMPOR method to eliminate batch effects between the two cohorts and excluded proteins that had missing values in 50% of the samples. The final proteomic profiles contained 8,973 quantified proteins in cohort one and 11,247 in cohort two. We then merged the proteomic profiles from both cohorts based on UniProt IDs, resulting in a combined profile of 8,777 proteins. Due to residual batch effects, we reapplied the TAMPOR method (Supplementary Figures S1A,B). Finally, we used non-parametric bootstrap regression to adjust for covariate effects [age, sex, and post-mortem interval (PMI)] on protein abundance, which had minimal impact on the final proteomic profiles (Supplementary Figure S1C).

Because the clinical data for the cohorts did not include age of onset information, we defined EOAD cases as those patients who died before the age of 65 and were posthumously diagnosed with AD, in accordance with the EOAD definition. Ultimately, we selected the proteomic data of 11 EOAD cases and 10 controls for subsequent analysis. The demographic characteristics of these subjects are presented in Table 1.

**Table 1:** Participant descriptions.

	Control (*n* = 20)	EOAD (*n* = 11)	*P*-value
Age, mean yrs. (SD)	63.7 (10.0)	60.0 (4.2)	0.161
Sex, (Male/Female)	10/10	6/5	0.553
PMI, median (IQR)	7.0 (3.0–28)	4.0 (3.5–6.0)	0.002
APOE-e4, at least one allele (%)	4 (20%)	9 (81.8%)	0.001
GREAD Score, mean (SD)	0.0 (0.0)	3.0 (0.0)	0.000
BRAAK Score, mean (SD)	1.5 (0.5)	6 (0.0)	0.000

### 3.2 Identification of differentially expressed proteins in EOAD brain tissues

**Figure 2:**
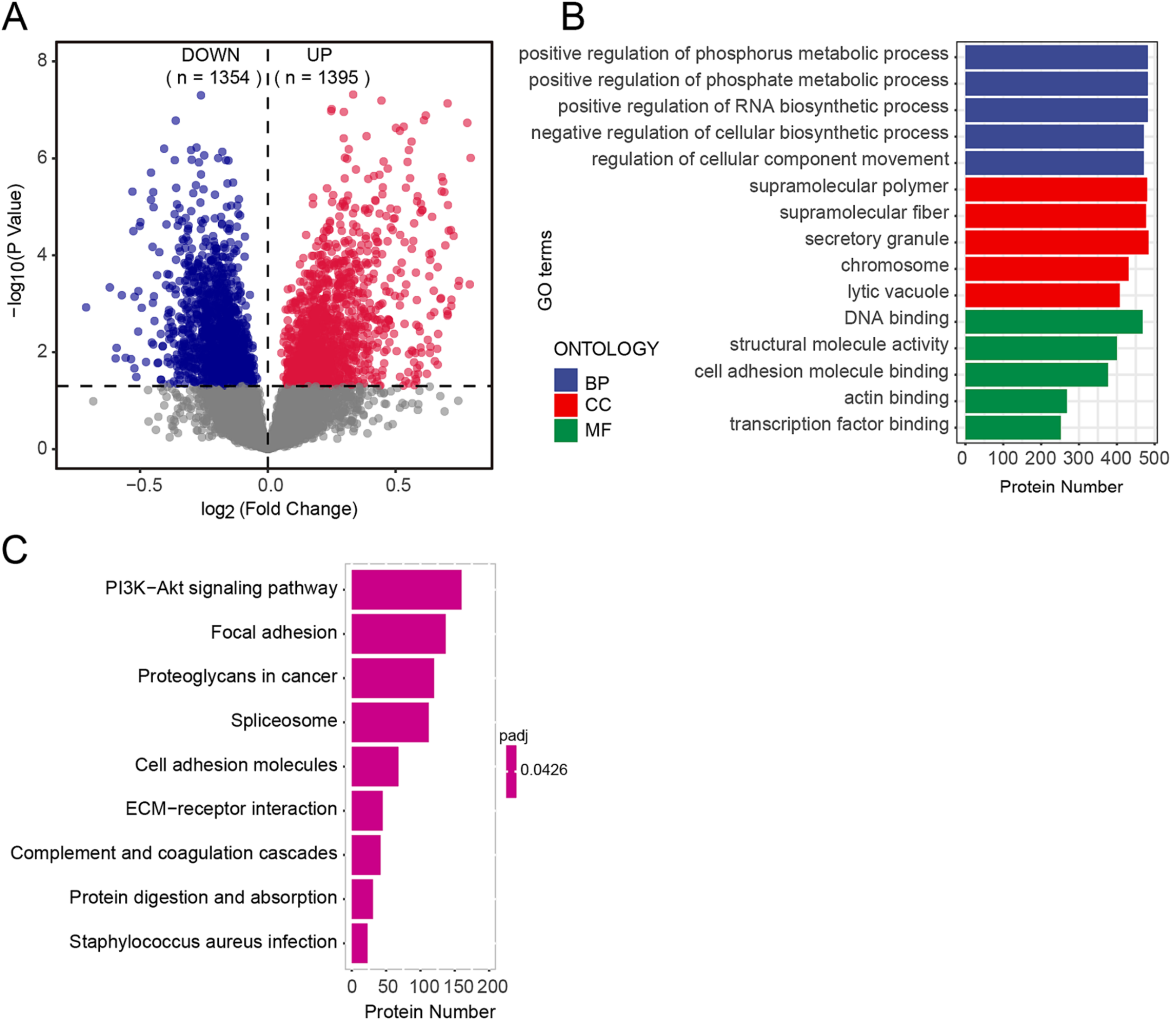
Proteomic characteristics of EOAD brain tissues. **(A)** Differentially expressed proteins were analyzed between groups within the cohort using volcano plots. *p*-values were calculated using students’ *t* test. A bold horizontal dashed line indicates the adjusted *p*-value threshold of <0.05. The number of significantly downregulated (blue) and upregulated (red) proteins is displayed at the top. **(B,C)** GO enrichment **(B)** and pathway enrichment **(C)** by GSEA.

We conducted Student’s *t*-tests to evaluate differential protein expression between the EOAD and control groups. The analysis revealed significant alterations in protein abundance in the EOAD group, with a total of 2,749 proteins showing significant changes (*p* < 0.05). Of these, 1,395 proteins exhibited significantly increased abundance, while 1,354 proteins showed significantly decreased abundance, as illustrated in Figure 2A. When applying a more stringent threshold of *p* < 0.0001, 255 proteins were identified as highly differentially expressed, with 159 proteins showing increased abundance and 96 proteins showing decreased abundance.

To account for the possibility that some proteins may influence the onset and progression of EOAD without displaying significant differential abundance, we performed GSEA on all detected proteins. The corrected *p*-values for enriched terms were all below 0.5. The results revealed enrichment in a total of 529 Gene Ontology (GO) terms. Specifically, biological processes (GO-BP) were primarily associated with phosphate metabolism, molecular functions (GO-MF) were predominantly related to DNA binding, and cellular components (GO-CC) were significantly enriched in supramolecular polymers (Figure 2B). Enriched pathways included the PI3K-Akt signaling pathway, complement and coagulation cascades, protein digestion and absorption, and staphylococcus aureus infection, among others (Figure 2C). Our findings highlight significant changes in the protein profile of EOAD brain tissue compared to the control group.

### 3.3 Construction of co-expressed protein networks

**Figure 3:**
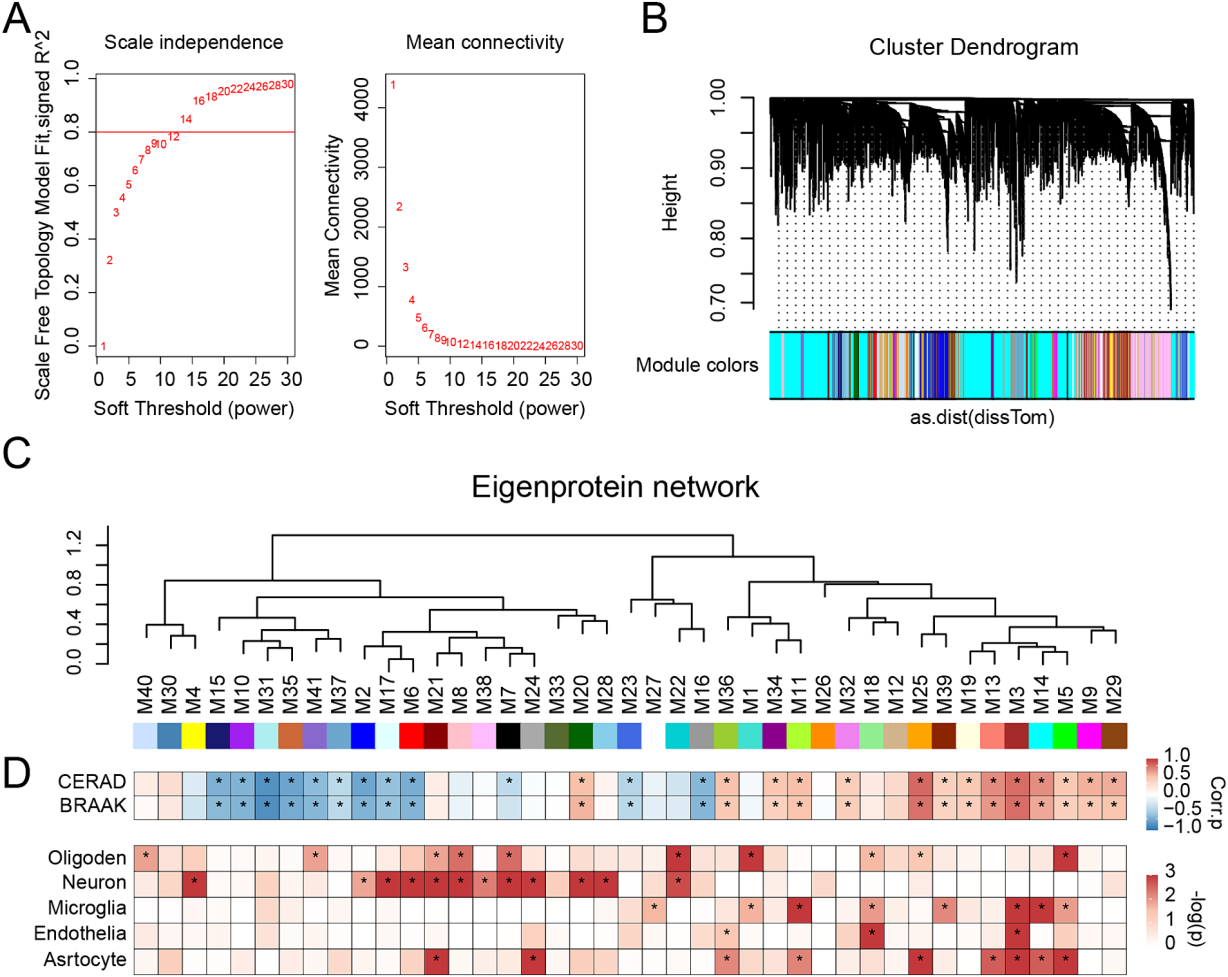
Protein network analysis of EOAD brain tissues. **(A)** Soft threshold (power = 13.5) and mean connectivity (R^2^ = 0.8) in the cohort. **(B)** Protein hierarchy tree-clustering diagram. The diagram represents different proteins horizontally and the correlation between them vertically. The lower the branch, the stronger the protein correlation within the branch. **(C)** The protein co-expression network consists of 41 protein modules, composed of 5,612 proteins from two cohorts. **(D)** Biweight mid-correlation (BiCor) analysis was conducted to assess the relationship between module eigenproteins and neuropathological features of AD (top), including CERAD scores and Braak staging. The strength of positive correlations is represented in red, while negative correlations are depicted in blue, with asterisks indicating statistical significance (*p* < 0.05). Cell-type associations for each protein module were evaluated using hypergeometric Fisher exact test (FET) (bottom). *p*-values resulting from FET were adjusted using the Benjamini-Hochberg (BH) method. The intensity of red shading reflects the degree of cell type enrichment, with asterisks indicating statistically significant differences (*p* < 0.05).

We employed the WGCNA algorithm to analyze the detected proteins in the brain, clustering their expression profiles into modules with similar patterns. The soft-thresholding power was set to 13.5 (R^2^ = 0.8) based on our calculations (Figure 3A). We then constructed the Topological Overlap Matrix (TOM) and merged the modules using hierarchical clustering and dynamic tree cutting (Figure 3B). Ultimately, 5,612 proteins were included, forming 41 distinct protein modules (Figure 3C), which were sorted and numbered according to the number of proteins in each module. The largest module contained 603 proteins, while the smallest had 37.

We calculated the eigenprotein for each module (i.e., the first principal component of the module proteins) and correlated it with neuropathological features of AD, specifically amyloid plaque deposition (CERAD score) and neurofibrillary tangles (BRAAK staging) (Figure 3D). Of the modules, 26 showed significant associations with the CERAD score (*p* < 0.05), with 12 exhibiting negative correlations and 14 positive correlations. Similarly, 25 modules were significantly associated with BRAAK staging (*p* < 0.05), with 11 showing negative correlations and 14 positive correlations.

Given that co-expression changes in brain proteins are often driven by alterations in cell types, we assessed the cellular nature of each module by identifying enrichment for cell-type-specific protein markers (Figure 3D). We found that 10 modules were associated with oligodendrocytes, 12 with neurons, 8 with microglia, 3 with endothelial cells, and 9 with astrocytes. In summary, our network efficiently detects modules associated with AD.

### 3.4 Identification of core module and key proteins

**Figure 4:**
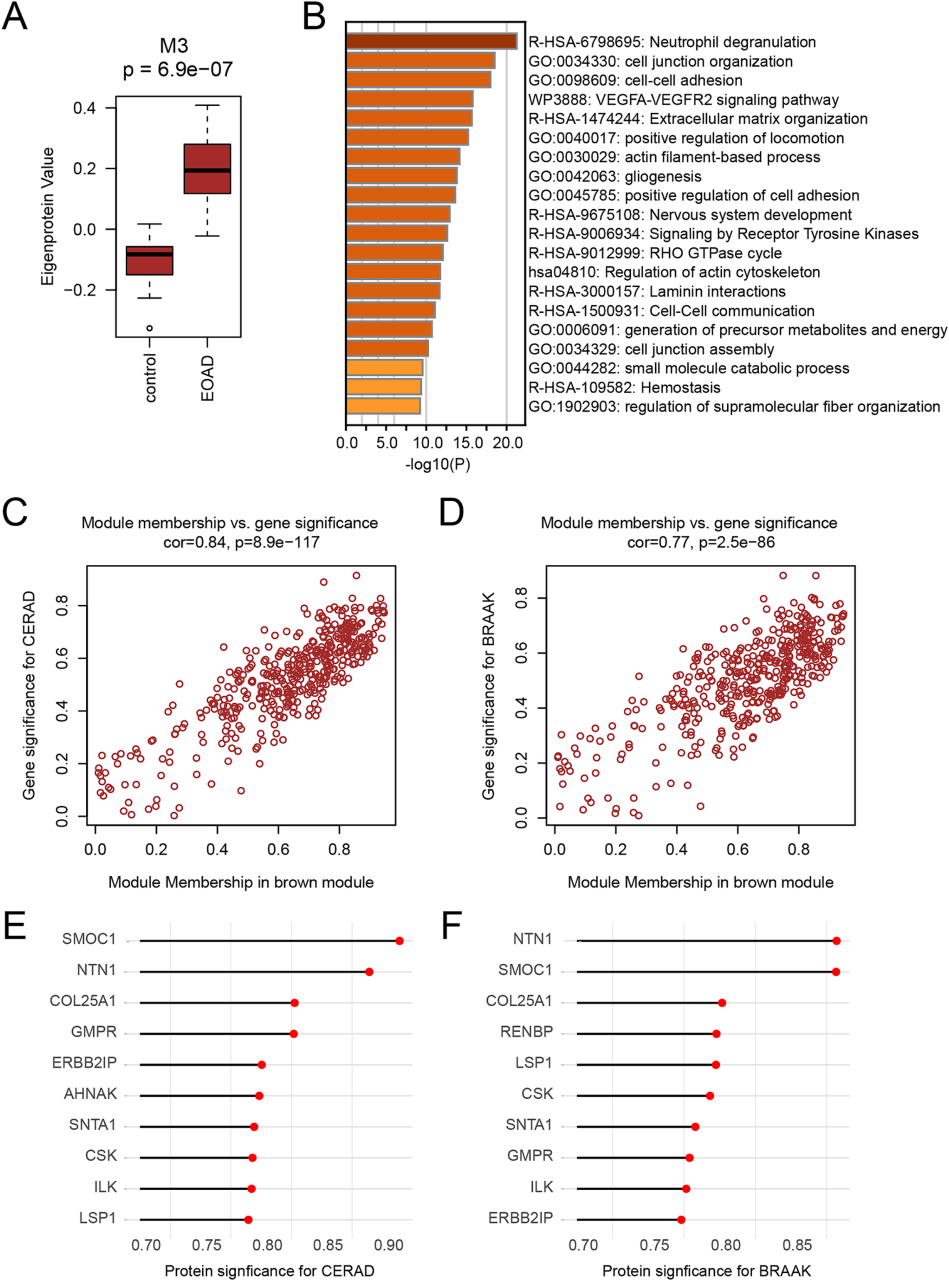
Identification of key proteins for EOAD **(A)** Boxplot show the level of eigenprotein between the CN and EOAD groups of M3. Student’s *t* test was used for comparison between the two groups. **(B)** Functional enrichment analysis of M3 module protein. Functional enrichment was performed using Metascape. For analysis, the top 20 most significantly enriched items were selected for presentation. Darker colors indicate a higher degree of enrichment. **(C,D)** Correlation of M3 module with CERAD score **(C)** and BRAAK staging **(D)**. Correlation coefficients and p-values were calculated by Pearson correlation analysis. **(E,F)** The protein significance of CERAD score **(E)** and BRAAK stage **(F)** in M3 module ranked in the top 10 proteins, respectively.

We performed a comparative analysis of the eigenproteins within each module. Notably, the M3 module was identified as the core module due to the most significant changes observed in its eigenprotein (Figure 4A). Functional enrichment analysis of the M3 module highlighted its primary involvement in neutrophil degranulation and the VEGFA-VEGFR2 signaling pathways (Figure 4B). Additionally, proteins within the M3 module showed a strong positive correlation with CERAD score and BRAAK staging, suggesting their potential role in promoting AD pathology progression (Figures 4C,D). We identified the top 10 proteins based on the absolute values of their significance for both CERAD scores and BRAAK staging as key proteins (Tables 2, 3). Among these, 9 proteins overlapped: SMOC1, NTN1, COL25A1, GMPR, ERBB2IP, SNTA1, CSK, ILK, and LSP1 (Figures 4E,F). These proteins are also among the most significantly different in EOAD compared to LOAD. Except for ERBB2IP and LSP1, the remaining seven proteins have been documented in AD research, confirming the reliability of our screening process (Tong et al., 2010; Georgakopoulos et al., 2011; Wyssenbach et al., 2016; Liu et al., 2018; Bai et al., 2020; Harrison et al., 2020; Wang et al., 2020). This suggests that ERBB2IP and LSP1 may also influence the progression of EOAD.

**Table 2:** Protein significance and module membership of top 10 proteins for CERAD in brown module.

	PS.CERAD	p.PS.CERAD	MMbrown	p.MMbrown
SMOC1|Q9H4F8–2	0.914215576	6.71 × 10^−13^	0.855946868	8.36279 × 10^−10^
NTN1|O95631	0.889272802	2.3 × 10^−11^	0.748725451	1.27026 × 10^−6^
COL25A1|Q9BXS0	0.82764751	9.3 × 10^−9^	0.842563444	2.77008 × 10^−9^
GMPR|P36959	0.826932818	9.83 × 10^−9^	0.935039441	1.36332 × 10^−14^
ERBB2IP|Q96RT1–8	0.800532891	6.42 × 10^−8^	0.921758006	1.85543 × 10^−13^
AHNAK|Q09666	0.798483113	7.34 × 10^−8^	0.943818931	1.75781 × 10^−15^
SNTA1|Q13424	0.79427638	9.62 × 10^−8^	0.783797625	1.83809 × 10^−7^
CSK|P41240	0.792862739	0.000000105	0.850261079	1.41077 × 10^−9^
ILK|A0A0A0MTH3	0.792135471	0.00000011	0.86910486	2.2781 × 10^−10^
LSP1|P33241-3	0.789544893	0.000000129	0.684760786	2.14708 × 10^−5^

PS, protein significance; MM, module membership.

**Table 3:** Protein significance and module membership of top 10 proteins for BRAAK in brown module.

	PS.BRAAK	p.PS.BRAAK	MMbrown	p.MMbrown
NTN1|O95631	0.881952961	5.55 × 10^−11^	0.748725451	1.27 × 10^−6^
SMOC1|Q9H4F8–2	0.881580767	5.79 × 10^−11^	0.855946868	8.36 × 10^−10^
COL25A1|Q9BXS0	0.801734896	5.93 × 10^−8^	0.842563444	2.77 × 10^−9^
RENBP|P51606	0.797731053	7.71 × 10^−8^	0.851002472	1.32 × 10^−9^
LSP1|P33241-3	0.79739162	7.88 × 10^−8^	0.684760786	2.15 × 10^−5^
CSK|P41240	0.793245815	1.03 × 10^−7^	0.850261079	1.41 × 10^−9^
SNTA1|Q13424	0.782983503	1.93 × 10^−7^	0.783797625	1.84 × 10^−7^
GMPR|P36959	0.778916225	2.46 × 10^−7^	0.935039441	1.36 × 10^−14^
ILK|A0A0A0MTH3	0.776594576	2.81 × 10^−7^	0.86910486	2.28 × 10^−10^

PS, protein significance; MM, module membership.

### 3.5 Validation of key proteins in AD model

In the proteomic analysis of our cohort, ERBB2IP and LSP1 were found to be significantly upregulated in the EOAD group compared to the control group (Supplementary Figure S3). Due to the limited availability of human brain tissue samples from EOAD patients, we validated the expression of ERBB2IP and LSP1 using a mouse model of AD. Specifically, we employed 5xFAD mice, which carry human APP and PSEN1 genes with five AD-related mutations, enabling early onset of amyloid deposition. We examined 3-month-old, 6-month-old, and 10-month-old 5xFAD mice alongside their wild-type (WT) littermates and performed immunoblotting analysis on their brain tissues.

**Figure 5:**
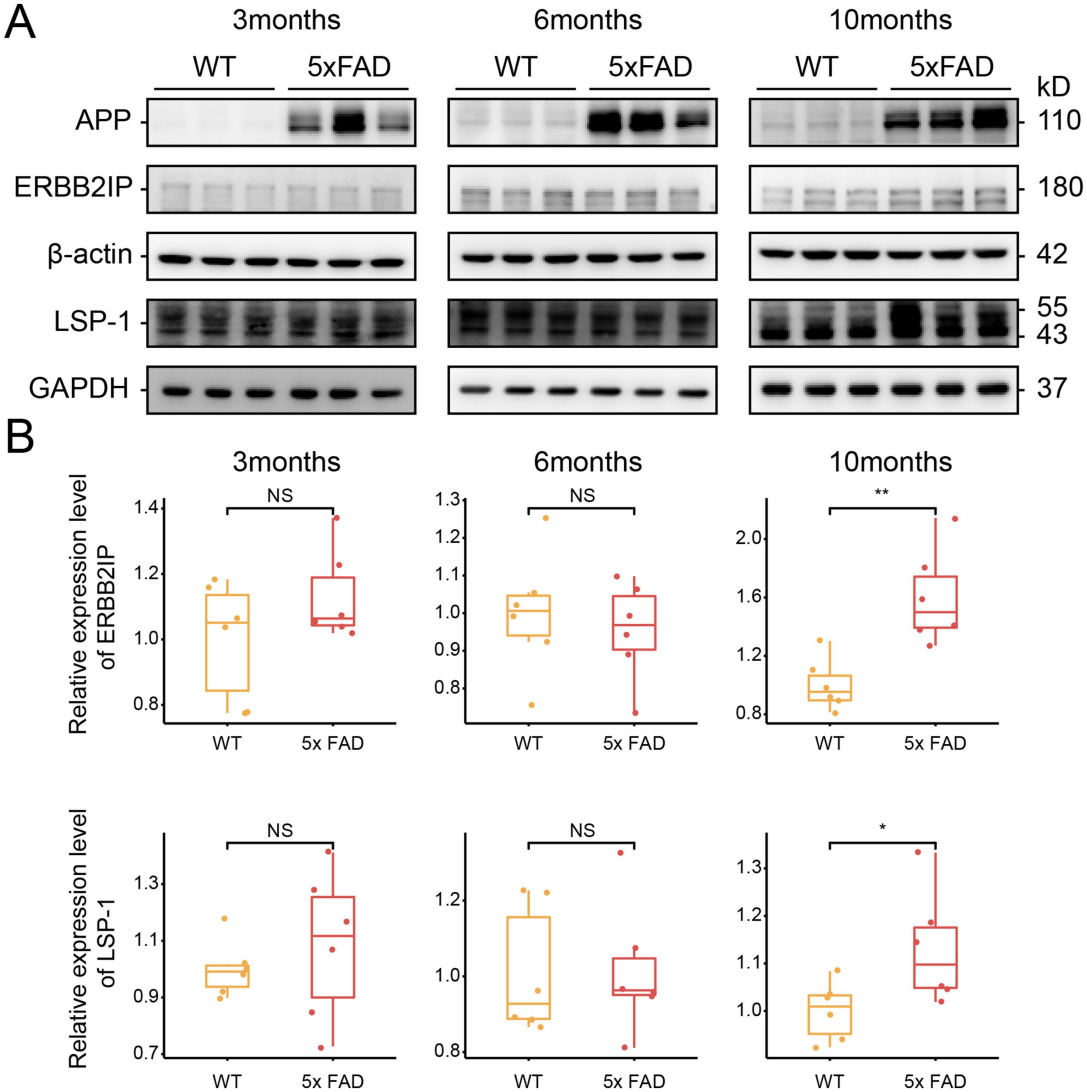
Expression of key proteins in 5xFAD mice brain tissue. **(A)** Representative immunoblots of ERBB2IP and LSP1 in the brain tissue. Internal protein was normalized to β-actin or GAPDH. **(B)** Quantification of ERBB2IP and LSP1 in the brain tissue. * means a *p*-value ≤0.05, **p means a *p*-value ≤0.01 (Students’ *t* test, *n* = 6 per each group).

Our results revealed no significant differences in the expression of ERBB2IP and LSP1 between 3-month-old and 6-month-old 5xFAD mice and their WT counterparts. However, in 10-month-old 5xFAD mice, the expression levels of both ERBB2IP and LSP1 were markedly increased (Figures 5A,B). This observation aligns with the proteomic data obtained from human brain tissues. In summary, these findings suggest that ERBB2IP and LSP1 may be involved in the late-stage progression of EOAD.

## 4 Conclusion

EOAD is a rare condition with notable heterogeneity, which may differ from LOAD in terms of etiology and pathogenesis. Proteomic studies of AD brain tissue seek to uncover the underlying biological processes and molecular mechanisms. However, no proteomic studies have specifically focused on EOAD brain tissue to date. In this study, we performed an integrated analysis of two AD cohorts, encompassing a total of 11 EOAD patients, to preliminarily investigate the molecular changes and biological processes in the brains of individuals with EOAD.

In this study, we observed significant changes in the protein profiles of EOAD brain tissue compared to controls. We constructed a protein co-expression network through WGCNA. We found that the core module most closely associated with the disease reflects biological processes of neutrophil degranulation and the VEGFA-VEGFA2 signaling pathway. Neutrophil degranulation has not received much attention in previous proteomic studies of AD (Wu et al., 2020). Neutrophils release various toxic molecules—such as reactive oxygen species, myeloperoxidase, glucosidase, proteases, and antimicrobial peptides—when activated by inflammation or infection (Kruger et al., 2015). This finding suggests a significant inflammatory response in the brains of individuals with EOAD. In AD model mice, Aβ can induce LFA-1 integrin-mediated cytoskeletal changes in endothelial cells, facilitating neutrophil infiltration into the brain parenchyma, a phenomenon not observed in wild-type mice (Baik et al., 2014; Zenaro et al., 2015). Research indicates that myeloperoxidase produced by neutrophils may contribute to oxidative stress in the brain vasculature, compromise the blood–brain barrier, and thereby accelerate AD progression (Smyth et al., 2022). Additionally, the expression of VEGFA in the brains and vascular systems of individuals with AD is complex and dysregulated. While VEGFA signaling can lead to capillary blockage and abnormal blood–brain barrier permeability, it may also facilitate A*β* clearance from the vasculature by promoting vascular repair in the AD brain (Chiarini et al., 2010; Ali et al., 2022).

We also identified two key proteins, ERBB2IP and LSP1, within the core module that have not been previously reported in AD. We validated their expression levels in an AD mouse model. Both proteins have been implicated in inflammatory responses in prior studies. ERBB2IP, a member of the epidermal growth factor receptor family (Borg et al., 2000), functions as a negative regulator and is involved in several cell signaling pathways, including the MAP kinase pathway, NF-κB signaling pathway, Ras–Raf–ERK signaling pathway, and TGF-β signaling pathway (Huang et al., 2003; McDonald et al., 2005; Dai et al., 2006; Dai et al., 2007). Research has demonstrated that ERBB2IP plays a crucial role in inflammatory diseases. A deficiency in ERBB2IP impairs NOD2-mediated NF-κB activation, while its overexpression in mouse embryonic fibroblasts significantly inhibits MDP-induced production of pro-inflammatory cytokines (Jang et al., 2021). Additionally, in a mouse colitis model, ERBB2IP expression in colonic tissues was notably reduced, and ERBB2IP-deficient mice exhibited increased susceptibility to intestinal inflammation (Shen et al., 2018). ERBB2IP can also mitigate the activation of the NLRP3 inflammasome and inhibit microglial pyroptosis, thereby reducing neuroinflammation associated with sepsis-related brain syndrome (Jing et al., 2022).

LSP1 is predominantly expressed in monocytes, macrophages, neutrophils, and endothelial cells (Jongstra and Davis, 1988; Jongstra et al., 1988; Pulford et al., 1999). Although the exact function of LSP1 remains not fully understood, it is known to play a crucial role in leukocyte chemotaxis during organ inflammation (Wang et al., 2007). In mouse models, the absence of LSP1 has been shown to reduce endotoxin-induced acute lung inflammation and decrease neutrophil migration to the lungs (Le et al., 2015). Additionally, elevated levels of LSP1 are associated with impaired myosin activity in neutrophils and are also linked to T cell migration in rheumatoid arthritis (Hwang et al., 2015; Ihentuge and Csoka, 2022).

The expression of ERBB2IP and LSP1 is significantly elevated in EOAD; however, their specific roles in EOAD, as well as their potential mechanisms and functions, remain unclear and warrant further investigation. Considering that both proteins are linked to inflammatory responses in other diseases, and given that neuroinflammation is a major pathological feature of AD, we hypothesize that they may also be involved in neuroinflammation. Inhibiting their expression could potentially reduce the neuroinflammatory response and thereby mitigate the pathology and cognitive decline associated with AD.

This study has several limitations. Firstly, due to the rarity of EOAD, the sample size is relatively small, which may introduce bias into the results. Secondly, in the absence of human brain samples from EOAD patients, we validated the expression of the key proteins using AD mouse models. Future research is needed to confirm these findings in human brains with EOAD. Thirdly, due to dataset limitations, we were unable to compare the proteomic data of EOAD with that of LOAD, thus preventing us from elucidating the differences between EOAD and LOAD. Understanding these distinct or shared molecular mechanisms may offer valuable insights into both diseases. In the future, we aim to include brain tissue from patients with confirmed EOAD and LOAD for proteomic analysis to elucidate their molecular differences. Finally, further *in vivo* and *in vitro* experiments are needed to determine whether these two key proteins impact AD pathophysiology or cognitive function, and to explore their biological functions.

In summary, we analyzed proteomic data from human brain tissue in cases of EOAD. This analysis allowed us to construct a protein co-expression network and identify biological processes and molecular pathways associated with EOAD. Within the core module, we pinpointed two key proteins, ERBB2IP and LSP1, that may be implicated in the progression of EOAD. These preliminary findings suggest that these proteins could serve as potential therapeutic targets for EOAD.

## Data Availability

Publicly available datasets were analyzed in this study. This data can be found here: syn20821165.

## Supplementary material

SUPPLEMENTARY FIGURE S1 Batch correction of the cohort. **(A-B)** Batch correction was performed within cohort one **(A)** and cohort two **(B)**. Batch correction was conducted between the merged protein expression profiles from cohort one and cohort two.

SUPPLEMENTARY FIGURE S2 Principal component analysis. Principal component analysis was conducted on protein expression profile of the merged cohort prior to regression analysis for covariates including age, sex, and postmortem time interval (PMI).

SUPPLEMENTARY FIGURE S3 Key proteins in the EOAD brain were significantly increased in the merged cohort. Boxplots show ERBB2IP and LSP1 protein levels in the control and EOAD groups. Asterisks represent the significance level of the Student's *t*-test: **** means a *p*-value ≤0.0001.
