# Supplementary figures and images for: Identification of key proteins in early-onset Alzheimer’s disease based on WGCNA
Source: Front Aging Neurosci. 2024 Oct 9;16:1412222. doi: 10.3389/fnagi.2024.1412222 (PMC11496171; doi:10.3389/fnagi.2024.1412222)

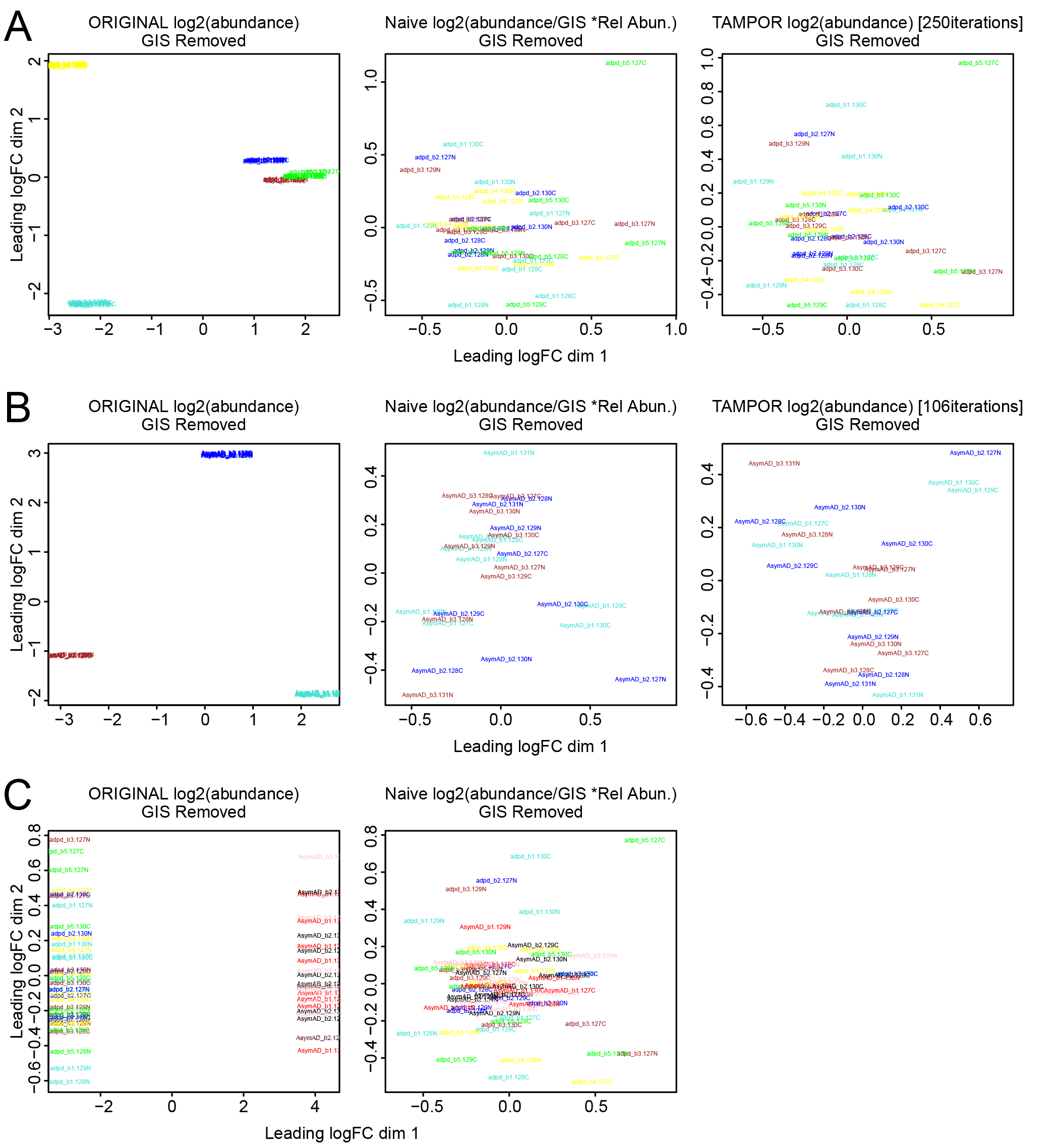

Supplement: SUPPLEMENTARY FIGURE S1 — Batch correction of the cohort. (A-B) Batch correction was performed within cohort one (A) and cohort two (B). Batch correction was conducted between the merged protein expression profiles from cohort one and cohort two. [file Image_1.tif]

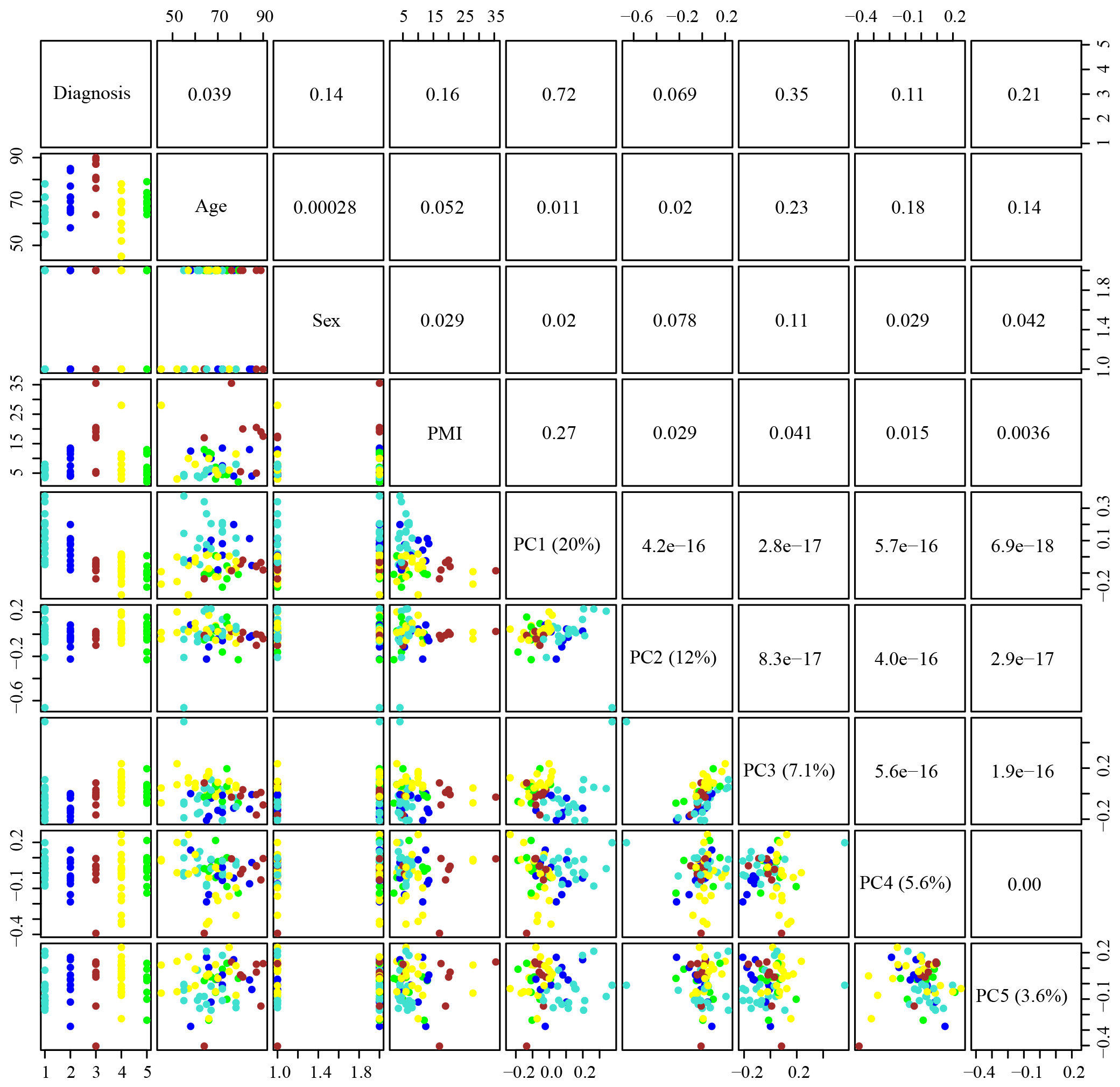

Supplement: SUPPLEMENTARY FIGURE S2 — Principal component analysis. Principal component analysis was conducted on protein expression profile of the merged cohort prior to regression analysis for covariates including age, sex, and postmortem time interval (PMI). [file Image_2.TIF]

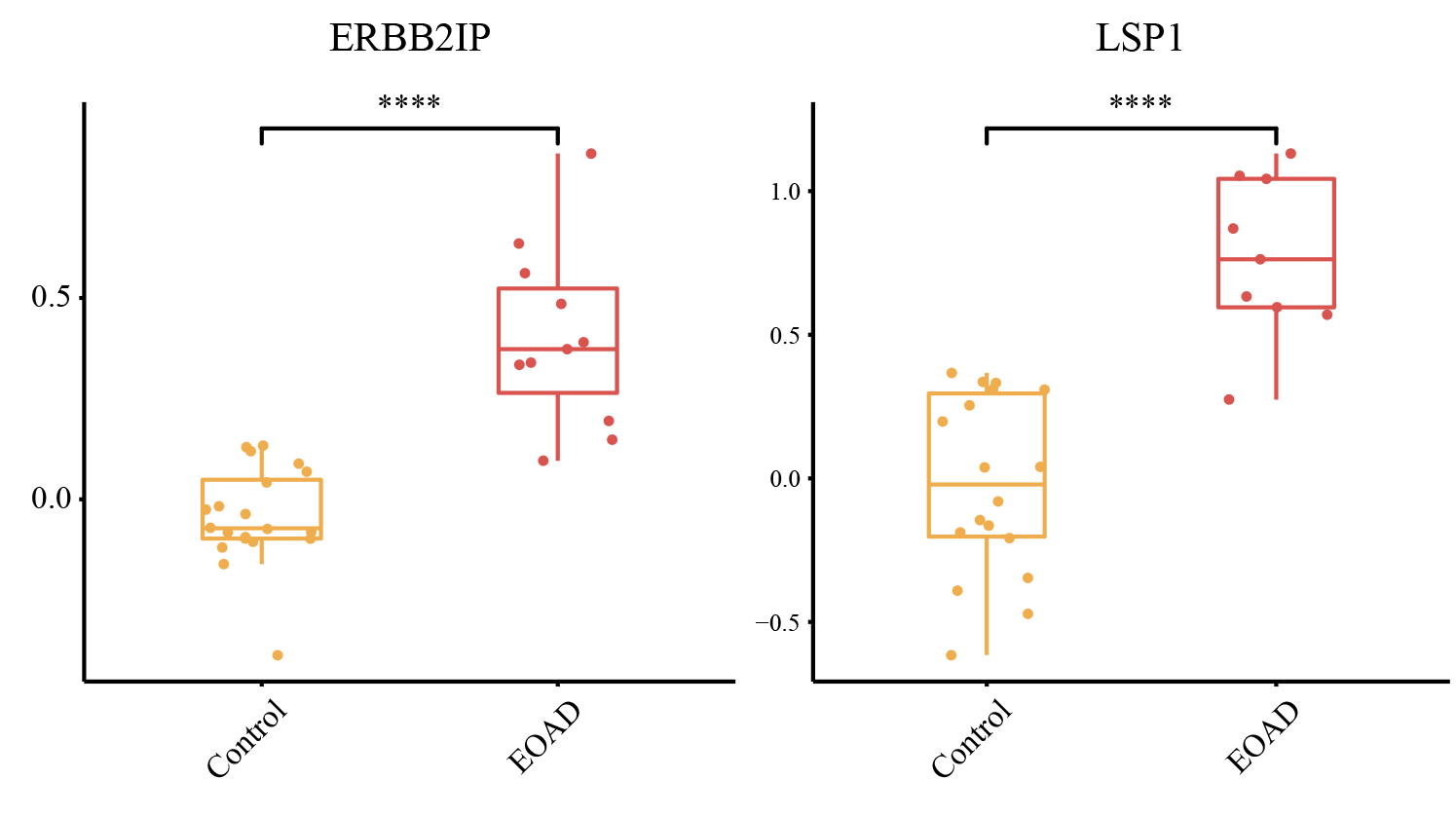

Supplement: SUPPLEMENTARY FIGURE S3 — Key proteins in the EOAD brain were significantly increased in the merged cohort. Boxplots show ERBB2IP and LSP1 protein levels in the control and EOAD groups. Asterisks represent the significance level of the Student's t-test: **** means a p-value ≤0.0001. [file Image_3.TIF]
